# Effect of telehealth on glycaemic control: analysis of patients with type 2 diabetes in the Whole Systems Demonstrator cluster randomised trial

**DOI:** 10.1186/1472-6963-14-334

**Published:** 2014-08-06

**Authors:** Adam Steventon, Martin Bardsley, Helen Doll, Elizabeth Tuckey, Stanton P Newman

**Affiliations:** 1 The Nuffield Trust, London, UK; 2 Oxford Outcomes, Oxford, UK; 3 School of Health Sciences, City University London, London, UK

## Abstract

**Background:**

The Whole Systems Demonstrator was a large, pragmatic, cluster randomised trial that compared telehealth with usual care among 3,230 patients with long-term conditions in three areas of England. Telehealth involved the regular transmission of physiological information such as blood glucose to health professionals working remotely. We examined whether telehealth led to changes in glycosylated haemoglobin (HbA1c) among the subset of patients with type 2 diabetes.

**Methods:**

The general practice electronic medical record was used as the source of information on HbA1c. Effects on HbA1c were assessed using a repeated measures model that included all HbA1c readings recorded during the 12-month trial period, and adjusted for differences in HbA1c readings recorded before recruitment. Secondary analysis averaged multiple HbA1c readings recorded for each individual during the trial period.

**Results:**

513 of the 3,230 participants were identified as having type 2 diabetes and thus were included in the study. Telehealth was associated with lower HbA1c than usual care during the trial period (difference 0.21% or 2.3 mmol/mol, 95% CI, 0.04% to 0.38%, p = 0.013). Among the 457 patients in the secondary analysis, mean HbA1c showed little change for controls following recruitment, but fell for intervention patients from 8.38% to 8.15% (68 to 66 mmol/mol). A higher proportion of intervention patients than controls had HbA1c below the 7.5% (58 mmol/mol) threshold that was targeted by general practices (30.4% *vs.* 38.0%). This difference, however, did not quite reach statistical significance (adjusted odds ratio 1.63, 95% CI, 0.99 to 2.68, p = 0.053).

**Conclusions:**

Telehealth modestly improved glycaemic control in patients with type 2 diabetes over 12 months. The scale of the improvements is consistent with previous meta-analyses, but was relatively modest and seems unlikely to produce significant patient benefit.

**Trial registration number:**

International Standard Randomized Controlled Trial Number Register ISRCTN43002091.

## Background

As the population ages, more people are living with long-term conditions such as diabetes, putting pressure on healthcare systems. Policy makers are exploring ways to improve the efficiency of healthcare services, while increasing or maintaining the quality of the care provided [1]. Care of patients with diabetes typically aims to reduce the risk of complications while minimizing harms associated with therapy, and thus increase longevity and health-related quality of life. Glycaemic control as measured by glycosylated haemoglobin (HbA1c) is a major goal [2], but it remains difficult for many patients to achieve targeted levels [3].

Self monitoring of blood glucose is well-established as an important component of diabetes management for insulin-treated patients [4] and has also been found to be important in some studies of non-insulin dependent patients [5,6]. Recently, increasing attention has been paid to remote monitoring using telehealth devices, which enable physiological information, including blood glucose, to be sent on regular basis to healthcare professionals working remotely [7]. Telehealth might be more effective at improving glycaemic control than ‘usual care’ including standard self monitoring, as it involves goal setting, transmission of blood glucose information, and more immediate feedback from a healthcare professional [8]. From a policy perspective, telehealth has been attractive in part because the greater oversight and self care believed to be associated with telehealth might lead to fewer, expensive unplanned hospital admissions and over time fewer long-term complications [9].

In a meta-analysis of 12 trials, telehealth was associated with lower HbA1c levels than usual care [10], but the average sample size was small, at 216 patients. Over half of the research participants represented in the meta-analysis came from a single trial (the IDEATel trial), which recruited 1,665 participants from medically underserved areas of New York State. In this trial, the improvements in HbA1c associated with telehealth were sustained over five years, and amounted to 0.29% at the end of that period (3.2 mmol/mol) [11-13]. IDEATel, however, began recruiting participants in 2002 when telehealth was relatively new. It used interventions designed by the evaluation team and eligibility criteria that excluded some groups of patients, such as those under age 55. It is therefore of interest to know the effect of telehealth when it is provided to a broader set of patients as part of the routine delivery of healthcare. People implementing telehealth today will likely make different decisions to the IDEATel group, because they have access to commercially-provided telehealth equipment and they operate in a different context.

In order to address weaknesses in the evidence about telehealth and other assistive technologies, the Department of Health in England announced three large demonstration sites in 2006 [14]. These sites were tasked with implementing whole system redesign aimed at integrating care teams, supported by a broad class of assistive technologies. The resulting evaluation included a large cluster randomised trial to assess the additional benefit of telehealth within this context (the Whole Systems Demonstrator trial, or WSD). Because pre-existing telehealth trials tended to be small and heterogeneous [15], the WSD aimed to have greater generalisability with over 3000 patients. To accomplish this aim, a pragmatic design was used [16] to compare telehealth with usual care (described below). A parallel trial, conducted in the same demonstration sites but not discussed in this paper, assessed the impacts of telecare for people with social care needs [17,18].

The WSD evaluation found some indications that telehealth was associated with fewer unplanned (‘emergency’) hospitalisations and deaths than usual care among a population with diabetes, heart failure or chronic obstructive pulmonary disease [19]. However, there were no overall improvements in quality of life for patients [20], and no evidence of cost effectiveness at usual willingness-to-pay levels [21]. The effect of telehealth on clinical metrics such as HbA1c has not previously been reported, but it would help to clarify the potential mechanisms for the observed effects and the role that telehealth can play towards meeting important treatment goals.

While the current study tested whether telehealth led to changes in glycaemic control among the subset of WSD participants with type 2 diabetes, another strand of the evaluation is assessing the effect of telehealth on disease-specific quality of life for these patients. Existing qualitative studies have assessed the patient, professional, and organizational factors related to implementation [22-24].

## Methods

### Design of the Whole Systems Demonstrator trial

The WSD was designed as a pragmatic trial to assess the impact of telehealth when introduced as part of the routine delivery of care. Unlike explanatory trials that compare highly-specified interventions in controlled situations on tightly defined cohorts of patients, pragmatic trials often use broad eligibility criteria to enrol patients, test flexible interventions as practiced by typical (not expert) practitioners, and make comparisons with usual care [25]. Therefore, an important part of the evaluation design was to leave aspects of the design of the telehealth interventions to local teams. The protocol for the trial and evaluation has already been published [14], along with a detailed description of the interventions (Web Appendix [21], and Web Appendix 2 [20]). We summarise the main features below.

The Department of Health selected the WSD sites in 2006 through a competitive process because (a) they were considered the most likely to succeed in scaling up remote care as part of a whole system redesign, and (b) they were considered representative of the range of local health and social care systems in England [22]. Cornwall, Kent and Newham in East London were chosen. In order to recruit suitably large numbers of patients, and because individual randomisation was unlikely to be acceptable to stakeholders, cluster randomisation was used [14]. Thus, all general practices in the three demonstration sites were invited to participate by site teams, and the general practices that accepted the invitation were randomised according to a centrally-administered minimisation algorithm to provide patients for either telehealth or usual care.

Patient inclusion criteria specified only age 18 years or over, plus a diagnosis of diabetes, chronic obstructive pulmonary disease or heart failure. Patients with these diagnoses were sourced from the existing lists of patients registered at participating general practices, using routine primary and secondary care data and referral letters from clinicians. Exclusion criteria related to the patient not understanding the instructions for the equipment (which were provided in English), or living in a home unsuitable for telehealth (for example, with inadequate telephone line connection). Eligibility was not conferred on the basis of a formal clinical assessment of disease severity, and patients with additional co-morbidities (in addition to the targeted long-term conditions) were not excluded from participation.

Once potentially eligible patients had been identified, they were written to at home and asked for their permission to share data with the research team so that their eligibility could be confirmed. Thus, all participants signed a consent form to agree to their details being examined to establish their eligibility for the trial. Once eligibility had been established, they were visited at home, where they signed a further consent form to agree to participate in the trial. Treatment allocations for patients followed those of the general practices at which the patients were registered. Though patients were not told about these allocations until after consent had been given, the long period of recruitment and cluster-randomised method meant it was not always possible to blind those recruiting patients.

The target sample size for the trial was 3,000 patients, based on the numbers needed to detect a 17.5% change in the primary outcome (hospitalisation proportion) from the levels assumed for usual care (with an intra-cluster correlation of 0.001 and an assumed cluster size of 25) [19]. Patient recruitment began in May 2008 and was scheduled to end in September 2009, though a small number of patients were recruited later.

The current study was conducted on the subset of WSD patients who had diabetes as their ‘index condition’. In the WSD trial, these index conditions were assigned in order to limit the number of disease-specific questionnaires given to patients; where a patient had more than one of the targeted long-term conditions, one condition was selected using randomisation [20]. We further restricted the sample to patients with type 2 (rather than type 1) diabetes by applying codes to the diagnoses recorded in general practice data (codes given in Additional file 1).

### Interventions

The trial was not designed to investigate the effect of individual service configurations or technologies [14]. Rather, it sought to understand the effects of ‘telehealth’, as a class of technologies that was added to standard support and treatment, compared with standard care alone. Consistent with the pragmatic design, sites were intentionally left to design aspects of their own telehealth services, such that there was some scope to tailor the services to local needs and resources. However, in all sites, telehealth equipment included a base unit (freestanding or a television set top box) and a glucometer for diabetes, weighing scales for heart failure and pulse oximeters for chronic obstructive pulmonary disease. In addition, most intervention patients received blood pressure monitors regardless of their long-term condition. Patients with multiple conditions could receive several peripherals. The peripherals communicated with the base unit either wirelessly or by cable.

The telehealth equipment reminded participants to take physiological measurements at the same time each day for up to five days per week, with the frequency adjusted according to participants’ individual clinical histories. For example, participants with diabetes and well-controlled blood glucose were typically asked to take readings less frequently than participants whose blood glucose was poorly controlled. In addition to the telemonitoring aspect of the intervention, symptom questions (e.g., ‘how are you feeling today compared with yesterday?’) and educational messages were sent to participants, either via the telehealth base unit or via a set top box connected to a television, depending on the study site.

Readings and answers to symptom questions were sent to monitoring centres using store-and-forward technology and secure servers. These centres were staffed with trained support workers and either specialist nurses or community matrons, depending on the site. Incoming biometric readings were automatically transformed into a traffic light classification of clinical risk, using thresholds (set initially on the basis of clinical guidelines or by a clinician responsible for the patient’s care) and algorithms that differed between sites. Urgent readings (‘red flags’) were reviewed and responded to daily, while other readings could also be reviewed to identify deteriorations in health. The scope of nurses to respond to the information from patients varied according to their role, with community matrons tending to have greater scope than monitoring centre nurses to initiate clinical interventions, such as a home visit or medicine titration within certain limits. However, all nurses could evaluate the patient, offer advice on disease management and refer patients to other healthcare professionals, such as general practitioners. Monitoring nurses were also able to transmit health related questions, messages, or videos to educate patients on their conditions, using the telehealth base unit or set top box.

The Department of Health provided funding to the demonstration sites for the telehealth interventions, as well as project manager support for the implementation of telehealth. Telehealth patients were not charged for using the telehealth services (including calls to the monitoring teams), but they were expected to have a telephone line and electricity.

Control patients were not provided with any specific support as part of their participation in the trial, though a subset participated in nested questionnaire studies [20,21]. They received usual care for the study sites, which excluded telehealth but for some patients included self-monitoring of blood glucose as agreed with their clinician. Control patients were offered telehealth at the end of the 12-month trial period if they were still eligible at that point.

### Collection of data on HbA1c

The original trial protocol specified that HbA1c would be collected at baseline, 3-month follow-up, and 12-month follow-up. During the course of the trial, however, practical issues with the complex, community-based recruitment procedure were identified that meant that the protocol was revised so that bespoke clinical measures were no longer collected. Instead, an analysis of the electronic medical record was planned. In England, these electronic records are used to calculate general practice entitlements to performance-related pay under the Quality Outcomes Framework (QOF). At the time of the trial, QOF criteria included the maintenance of codes for the diagnosis of diabetes, together with regular recording of HbA1c [26]. Although it was recognised that these data would have limitations (discussed below), it was expected that they would be more complete for diabetes than for heart failure or chronic obstructive pulmonary disease.

All general practices participating in the trial were asked to share event-level data from the electronic medical record for their registered adult patients [27]. Data were effectively anonymised (‘pseudonymised’) before they were transferred to the evaluation team, with patient identifiers removed and a unique patient identifier (the ‘NHS number’) encrypted. The encrypted NHS number was used to link the general practice data to trial data sets [17].

The use of routine data meant that it was necessary to exclude a small number of participants. First, we excluded patients who were not linked to the routine data because NHS numbers were not available for encryption. A second exclusion arose because our study focused on the period from two years before a patient was recruited in the trial to 12 months following that recruitment (or death, if sooner) – this was known as the ‘observation period’. We were concerned that, although all patients were registered at a participating general practice at the time of trial recruitment, they might have been registered at another general practice at some other point during the observation period. If this other general practice did not provide data for the evaluation (for example, because it did not participate in the trial), then we would have incomplete information about diagnosis codes and HbA1c. As a result, we restricted the analysis to patients who were always registered with one of the observed general practices during the observation period.

### Baseline characteristics

Baseline variables were derived using the general practice data (see codes in Additional file 1). Consistent with other strands of the evaluation [19], for the purpose of calculating variables, the trial start date was taken as the date of telehealth installation for intervention patients, and as the date of the initial project team visit for controls.

In cluster-randomised trials, selection bias is theoretically possible, either through systematic differences between practices in the control and intervention groups, or through similar differences at the individual level [28]. Following previous work [19], we present standardised differences as a summary measure of differences between groups at baseline; these were defined as the differences in sample means (or proportions), divided by the pooled standard deviation [29]. For continuous variables, we also present the variance ratio, defined as the variance in the intervention group divided by the variance among controls [30].

### Analysis of HbA1c frequency

We did not expect telehealth to alter the frequency with which HbA1c was recorded in general practice (as indicated by previous work [27]). To confirm this, we compared numbers of HbA1c readings during the 12-month trial period between treatment groups using chi-squared tests. We also tested whether readings were made uniformly across the trial period, using the Kolmogorov-Smirnov test and pooled data across both treatment groups.

### Comparison using repeated measures models

Analysis of HbA1c levels was conducted on an intention-to-treat basis, using all HbA1c readings recorded within the observation period. Analysis was conducted using a repeated measures linear regression model, which included adjustment factors (fixed effects) for:

• Time (to account for secular trends in HbA1c);

• Trial arm (to account for differences in HbA1c between treatment groups before the start of the trial); and

• The interaction of trial arm and period (to estimate the impact of allocation to the telehealth intervention on HbA1c during the trial period).

As HbA1c readings for the same individual at different time points will tend to be correlated, the model included a first-order autoregressive correlation structure. As patients at the same general practice will tend to have more similar HbA1c readings than patients at different general practices, the model included random effects at the general practice (cluster) level. Finally, the model included random effects for the time of the readings because these were not made on a fixed schedule.

### Comparison of average HbA1c before and after recruitment

In order to test the robustness of our results to the models used, we conducted a series of secondary analyses. These were done on a ‘completer’ basis and thus were restricted to the subset of study participants who had at least one HbA1c recorded during the 12-month trial period. Analyses were conducted at the patient level, summarising the HbA1c readings recorded for each individual during the trial period using the mean. Differences between the treatment groups were then assessed using mixed linear regression [31]. The model included random effects for the general practice. As well as doing unadjusted analysis, we also adjusted for a set of baseline characteristics in the regression. These were: last-recorded HbA1c prior to recruitment; age; sex; last-recorded blood pressure, cholesterol quartile and BMI quartile prior to recruitment; site (Cornwall, Kent or Newham); smoking status; co-morbid ischemic heart disease; and diabetes management (insulin or non-insulin). We conducted a range of sensitivity analyses with other model specifications. These are described in Additional file 2.

### Proportions of patients below thresholds

At the time of this trial, general practices in England had a specific goal to maintain glycosylated haemoglobin (HbA1c) below 7.5% (58 mmol/mol) for patients with diabetes. Therefore, we compared the proportion of intervention and control patients under this threshold, again averaging multiple readings for the same individual. Between-group differences were assessed using mixed logistic regression, with random effects for the general practice. We also used graphical methods to compare the distributions of HbA1c between intervention and control groups (again, averaged over the trial period). This was done using the HbA1c cut-off points adopted in a previous study, which used electronic data from general practices in the UK to examine the relationship between HbA1c and survival [32].

### Ethical approval

The Liverpool Research Ethics Committee (reference 08/H1005/4) approved the study in all of the participating sites.

## Results

**Figure 1:**
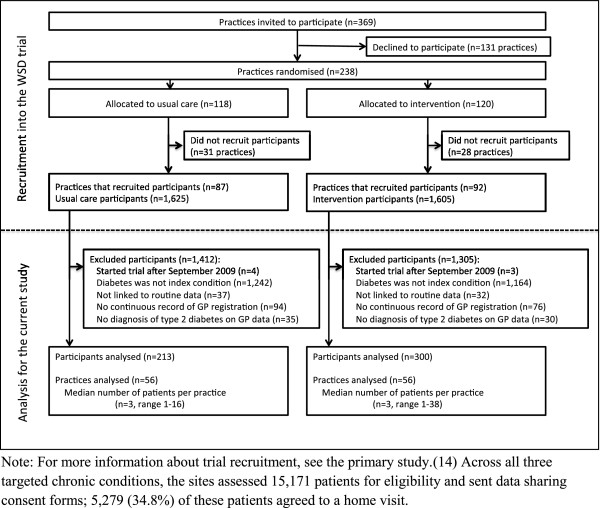
Flow of patients into the study.

The full WSD trial involved 3,230 patients from 179 general practices [19]. Of these, 817 patients had diabetes as their index condition and were recruited before 30 September 2009 (n = 379 control; 438 intervention). As previously stated, we excluded patients who were not linked to the routine data (n = 37 control; 32 intervention), did not have a continuous record of general practice registration (n = 94 control; 76 intervention), or did not have a confirmed diagnosis of type 2 diabetes within the general practice data (n = 35 control; 30 intervention). This left us with 513 patients with type 2 diabetes (n = 213 control; 300 intervention), from 112 general practices (Figure 1).

Prior to recruitment, the last-recorded HbA1c was 8.3% (67 mmol/mol) for control patients on average, compared with 8.5% (69 mmol/mol) for intervention patients (standardised difference 10.6, variance ratio 1.3) – see Table 1. A similar proportion in each group had been prescribed insulin in the year before recruitment (46.5% of control patients *vs.* 49.7% of intervention patients). Rates of prescription of sulphonylureas were also similar (41.3% of control patients *vs.* 40.3% of intervention patients), though a smaller proportion of control than intervention patients had been prescribed metformin (69.5% *vs.* 77.3%). Compared with the control group, the intervention group was larger (300 *vs.* 213), more likely to be female (46.7% of patients *vs.* 35.7%) and younger (mean age 63.9 years *vs.* 66.2).

**Table 1:** Baseline characteristics of study cohort (all data are proportions of patients unless otherwise specified)

	**Control (N = 213)**	**Intervention (N = 300)**	**Standardised difference (variance ratio)**
Site			
Cornwall	20.2	22.3	5.2
Kent	24.9	14.3	-26.8
Newham	54.9	63.3	17.2
Other targeted conditions			
COPD	11.3	16.3	14.7
Heart failure	12.7	13.0	1.0
Age			
Mean (SD) age in years	66.2 (11.9)	63.9 (13.0)	-18.2 (1.2)
<65 years	43.7	50.7	14.1
65-74 years	31.0	29.7	-2.9
75-84 years	21.1	16.3	-12.3
> = 85 years	4.2	3.3	-4.7
Female	35.7	46.7	22.5
Ethnicity			
White	55.4	51.7	-7.5
Other	33.3	30.3	-6.4
Unknown	11.3	18.0	19.1
Area-level deprivation score*			
Mean (SD)	34.2 (13.0)	36.7 (14.4)	18.3 (1.2)
First quartile	4.2	4.3	0.5
Second quartile	8.0	7.0	-3.7
Third quartile	23.9	18.7	-12.9
Fourth quartile	63.8	70.0	13.1
Mean Combined Model score (SD)**	0.25 (0.18)	0.24 (0.19)	-3.8 (1.2)
Ischemic heart disease	14.6	8.3	-19.6
Diabetes management in prior year			
Insulin	46.5	49.7	6.4
Sulphonylureas	41.3	40.3	-2.0
Metformin	69.5	77.3	17.8
Thiazolidinediones	9.4	12.3	9.5
Smoking status			
Never	42.3	46.3	8.2
Current	12.2	10.7	-4.8
Ex smoker	45.5	43.0	-5.1
Latest clinical readings (mean (SD))			
HbA1c	8.3 (1.7)	8.5 (1.8)	10.6 (1.3)
Body-mass index (BMI)***	30.3 (5.9)	31.8 (6.6)	24.2 (1.3)
Cholesterol****	4.3 (1.1)	4.3 (1.4)	2.5 (1.5)
Systolic blood pressure	134.4 (17.6)	132.1 (19.1)	-12.6 (1.2)
Diastolic blood pressure	74.3 (10.2)	75.0 (10.3)	6.3 (1.0)
Latest HbA1c < = 7.5%	35.7	33.3	-4.9
Mean (SD) health care contacts per patient in prior year			
Practice nurses	6.0 (7.5)	4.9 (7.7)	-15.0 (1.1)
General practitioner	8.5 (7.2)	9.2 (6.0)	10.2 (0.7)
Elective admissions	0.5 (1.2)	0.4 (0.8)	-15.7 (0.5)
Emergency admissions	0.5 (1.1)	0.5 (1.0)	-3.7 (0.7)
Outpatient attendances	6.7 (8.9)	5.8 (6.9)	-11.1 (0.6)
Emergency department visits	0.5 (1.1)	0.7 (1.3)	16.2 (1.2)
Hospital bed days	3.3 (6.4)	3.7 (9.3)	5.4 (2.1)

SD = standard deviation.

*Based on the Index of Multiple Deprivation, 2007, attributed to small areas of residence.

**Combined Model score is the estimated probability of emergency hospitalization within the 12-month trial period [33].

***N = 189 (control); 245 (intervention).

****N = 212 (control); 298 (intervention).

During the 12-month trial period, the majority of the 513 patients included in the study had at least one recorded HbA1c (95.3% of control patients and 93.0% of intervention patients). The mean number of HbA1c readings per patient during this period was 1.9 (median 2, range 0–7), with no difference in the distribution of number of readings between control and intervention groups (chi-square statistic 5.14, p = 0.64). Readings appeared to be recorded uniformly within the trial period (Kolmogorov-Smirnov statistic 0.028, p > 0.25).

The repeated measures analysis (conducted on the full sample of 513 patients) found that the intervention patients had lower HbA1c than control patients during the 12-month trial period, by 0.21% (2.3 mmol/mol) in favour of the intervention group. This difference was statistically significant at the 5% level, with a 95% confidence interval ranging from 0.04% (0.4 mmol/mol) to 0.38% (4.2 mmol/mol) (p = 0.013).

**Figure 2:**
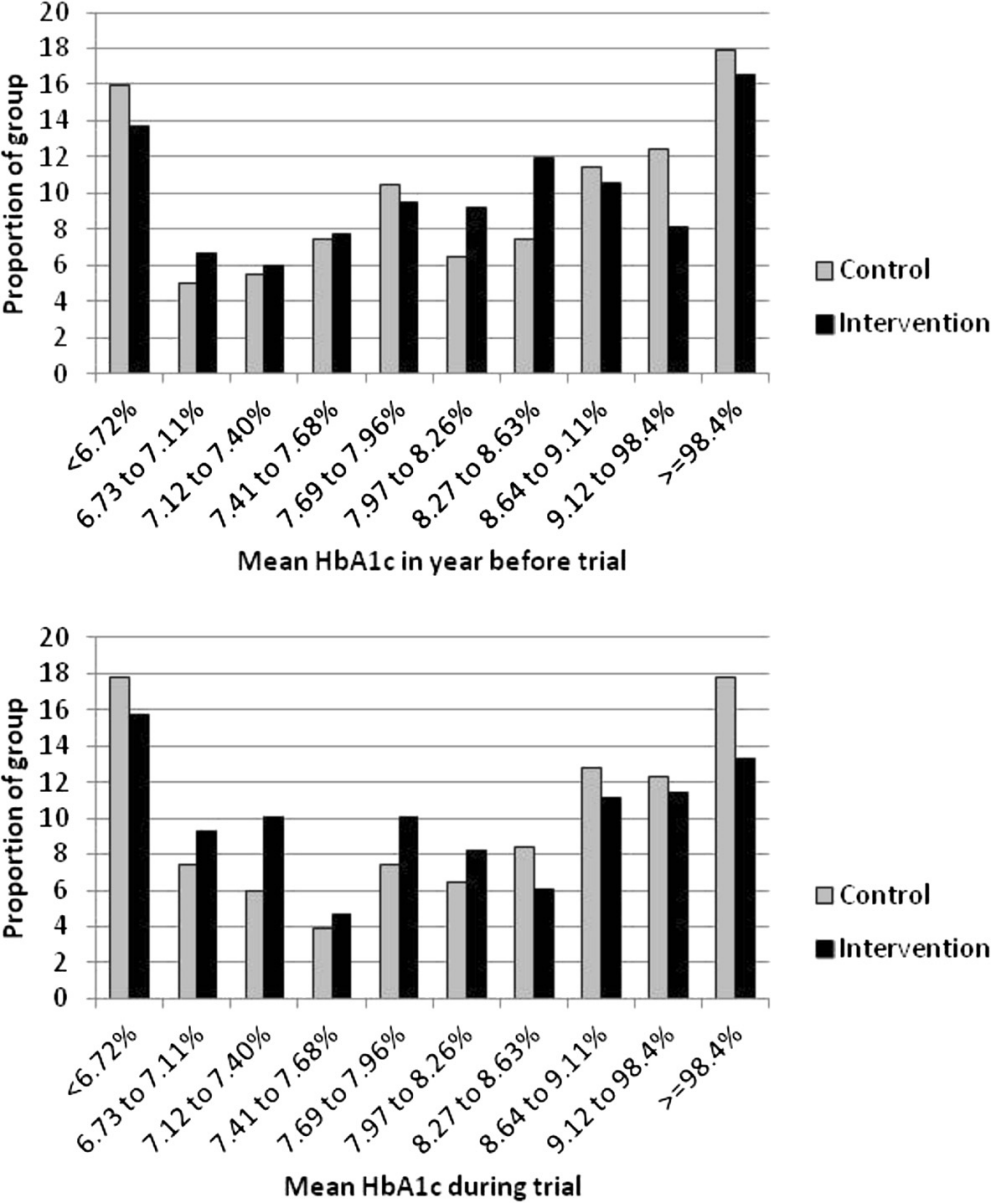
**Distribution of mean HbA1c during trial period.** Bands are based on Currie et al. (2010) [32].

Secondary analysis was restricted to the 457 patients with at least one HbA1c reading in the years before and after recruitment (Table 2). Among control patients, mean HbA1c was 8.41% (68 mmol/mol) during the year before recruitment, and 8.38% (68 mmol/mol) during the 12-month trial period. Mean HbA1c thus showed little change among control patients. However, it fell among intervention patients from 8.38% (68 mmol/mol) to 8.15% (66 mmol/mol). Differences between treatment groups were not statistically significant in the unadjusted analysis, though they reached significance when adjusting for baseline characteristics - see Table 2. Sensitivity analysis found that, although effect sizes did not depend heavily on the model specification, statistical significance was not always reached (Additional file 2).Compared with controls, a smaller proportion of the intervention group appeared to have high mean HbA1c during the trial period (Figure 2). During the 12-month trial period, 30.4% of control patients had mean HbA1c less than the 7.5% threshold targeted by general practices (58 mmol/mol), compared with 38.0% of intervention patients. This difference was not statistically significant in the unadjusted analysis (odds ratio 1.43, 95% CI, 0.94 to 2.17, p = 0.095), though it approached significance when adjusting for baseline characteristics (adjusted odds ratio 1.63, 95% CI, 0.99 to 2.68, p = 0.053).

**Table 2:** Secondary analysis of HbA1c (%) for patients with levels recorded within the year before the trial and within the year of the trial itself

	**Control group mean (standard deviation) (n = 191)**		**Intervention group mean (standard deviation) (n = 266)**		**Difference in mean HbA1c during trial (95% confidence interval)**	
	**Before trial**	**During trial**	**Before trial**	**During trial**	**Unadjusted**	Adjusted*
Mean HbA1c	8.41 (1.64)	8.38 (1.60)	8.38 (1.68)	8.15 (1.49)	-0.26	-0.30
					(-0.60, 0.07)	(-0.52, -0.07)
					p = 0.125	p = 0.009

*Adjusted for age; sex; last recorded HbA1c, blood pressure, cholesterol quartile and BMI quartile; site (Cornwall, Kent or Newham); smoking status; co-morbid ischemic heart disease; and diabetes management (insulin or non-insulin).

## Discussion

The Whole Systems Demonstrator trial tested the effect of telehealth in the context of whole system redesign in three areas of England. Its pragmatic design allowed for the recruitment of very large numbers of patients, making it the largest randomised controlled trial of telehealth conducted thus far. We found that, among a subset of 513 participants with type 2 diabetes, telehealth was associated with modestly lower HbA1c than usual care over a 12-month period, by around 0.21% (2.3 mmol/mol). Following recruitment, HbA1c fell from 8.38% to 8.15% (68 to 66 mmol/mol) among telehealth participants, whilst there was broadly no change among controls. Telehealth appeared to be associated with a higher proportion of patients meeting the 7.5% (58 mmol/mol) threshold that was targeted by general practices in England at the time of the trial, though this did not quite reach statistical significance (38.0% *vs.* 30.4% of patients, adjusted odds ratio 1.63, 95% CI, 0.99 to 2.68, p = 0.053).

### Strengths and weaknesses

The complexity of the recruitment processes and the size of the trial meant that we relied on routine electronic data for HbA1c, rather than bespoke data collection. Routine data offer promising opportunities for research [34], and this study offers one model for how routinely-recorded clinical information can be used in analyses of randomised controlled trials. One challenge was that the timing of the available HbA1c measurements was contingent on decisions made by patients and professionals, rather than, as in a conventional trial, specified by the trial protocol. If, for example, HbA1c tends to be taken in response to concerns about patient health, we may have disproportionately sampled HbA1c when it was higher. In assessing the potential bias from our data-collection approach, we were reassured that the intervention itself did not appear to lead to changes in the frequency of measurement. Further, results from our primary and secondary analyses were similar. As the secondary analysis averaged multiple readings recorded for each individual, they ensured that all individuals contributed equal weight to the analysis, regardless of the number of readings made for each one The similarity of the results of the analyses suggests that they were not biased towards patients who attended general practice most often. We note that our findings reflect the average HbA1c recorded over a 12-month period. We did not assess whether short-term effects on HbA1c (such as at three months) were larger than those at 12 months. This could have occurred, if, for example, patients adhered to the monitoring schedule more closely at the start than at the end of the trial.

Selection bias is recognised as a risk in cluster randomised trials, particularly when the individuals recruiting patients have foreknowledge about treatment allocations, as might sometimes have been the case in this trial. Reassuringly, we did not find large baseline differences between intervention and control groups in the primary study of all 3,230 patients [19]. In the current study, with smaller samples, larger differences were more likely to have occurred by chance. The most notable difference concerned the size of the treatment groups (300 patients for the intervention group *vs.* 213 patients for the control group). Furthermore, although general practices in the intervention and control arms typically provided similar numbers of patients for the current study (median 3 patients in each arm), some intervention practices supplied up to 38 patients, compared with up to 16 for control practices. This pattern was also not systematic across the wider trial, and indeed the primary study found differences in cluster sizes in the opposite direction [19].

To limit the impact of selection bias, we conducted analysis on an intention-to-treat basis and adjusted for important prognostic variables including age, smoking status, co-morbid ischemic heart disease, and diabetes management. Adjustment tended to make estimated effect sizes larger. The use of routine data meant that some covariates of interest were not available, in particular time since diagnosis, prior self-monitoring activity and micro-vascular complications. We therefore had to rely on randomisation to balance these characteristics between intervention and control groups. Although cluster randomisation has limitations, it reduced the scope for contamination between treatment groups as no general practice provided care for both telehealth and control participants. Individual randomisation would not have been acceptable to the stakeholders involved in this study [14].

Approximately 80% of the individuals invited to participate in the WSD trial refused to do so [19]. Such high rates of patient refusal are not unusual for telehealth trials [35], and by themselves suggest that telehealth might not always meet the perceived needs of patients and that many patients may be reluctant to change from their current mode of care [23,36]. A qualitative study explored the reasons given by patients for not participating in the WSD trial. These related to the requirements for technical competence and operation of the telehealth equipment; threats to identity, independence and self-care; and expectations and experiences of disruption to services [23]. One implication of the high refusal rate is that the included patients were probably not fully representative of the wider population with type 2 diabetes. Compared to cross-sectional studies of the diabetes population, the current study included a relatively high proportion of insulin-treated patients (49.7%) [3]. Also, baseline levels of HbA1c were higher in the current study than in IDEATel [11] (mean 8.5% *vs.* 7.4%, or 69 *vs.* 57 mmol/mol). Some comorbidity arose from the other two conditions targeted by WSD, namely heart failure (affecting 13.0% of intervention patients) and chronic obstructive pulmonary disease (affecting 16.3%). Overall, it seems likely that we studied a population with relatively less well-controlled diabetes. A formal, empirical assessment of the generalisability of the WSD trial is planned in relation to its primary endpoint of hospitalisation. This assessment will use observational data sets to compare the baseline characteristics and outcomes of WSD control patients with the baseline characteristics and outcomes of patients receiving usual care outside of the trial [37].

Usual care already consisted of a blend of different (non-telehealth) approaches to improving glycaemic control. Information was not available on rates of self-monitoring in the control group, but it would not have been universal [4]. Telehealth was implemented in the context of wider, whole-systems redesigns on-going in the sites. This redesign might have affected care delivered to both intervention and control patients, though a companion study found that the extent of integration achieved during the evaluation period was quite limited [22].

Although the WSD sites were diverse, they were selected based on their history of innovations in these areas of care. Furthermore, there was dedicated funding and project management for telehealth in this trial. Telehealth might have different effects in other settings, and important factors might include: which patients are recruited; the available staff and how they undertake their roles; other on-going approaches to managing long-term conditions; the local context, including financial incentives; integration between care teams; and the details of the telehealth and health information technology and monitoring systems. In general, the recruitment and monitoring protocols used in randomised controlled trials can affect outcomes for both intervention and control groups, for example through Hawthorne effects [38].

### Comparison with existing studies

Our findings are in line with previous meta-analyses of the effect of telehealth versus usual care. One of these meta-analyses examined 12 randomised controlled trials and found reductions in HbA1c of around 0.22%, or 2.4 mmol/mol (95% CI, 0.08 to 0.35%, or 0.9 to 3.8 mmol/mol) [10]. Another examined 15 trials and found smaller impacts (-0.10% or 1.1 mmol/mol) that did not reach statistical significance [39]. In comparison to these meta-analyses, larger effect sizes where found in a review of telehealth interventions using mobile phones for patients [40]. These interventions were associated with reductions in HbA1c of 0.8%, or 8.7 mmol/mol (95% CI, 0.5 to 1.1%, or 5.5 to 12.0 mmol/mol). Overall, the evidence suggests that interventions such as telehealth are, at best, associated with modest reductions in HbA1c compared with usual care [41].

A number of differences exist between WSD and the previous IDEATel trial, which found larger impacts on HbA1c [12]. For example, the telehealth services developed by the WSD sites were largely based in primary care [14], while IDEATel contained an element of case management under diabetologist supervision. A particular feature of WSD is that we left the development of telehealth services largely to the participating sites. Although the resulting plurality might be problematic for the purposes of replicating the specific interventions, in some ways it is the merit of a pragmatic trial as it means that our results reflect decisions made by local teams, thus increasing their generalisability to routine clinical practice.

## Conclusions

In this study, telehealth led to modest improvements in glycaemic control among people with type 2 diabetes as indicated by glycosylated haemoglobin (HbA1c), at least over 12 months. The improvements (0.21%, 95% CI, 0.04% to 0.38% - equating to 2.3 mmol/mol, 95% CI, 0.4 to 4.2) are consistent with previous meta-analyses.

Although further investigation is warranted, it seems doubtful that the improvements in HbA1c that we detected in the current study were sufficiently large to produce substantial patient benefit. Other interventions, which report reductions in complications from diabetes, have shown larger reductions in HbA1c than the current study. For example, the Diabetes Control and Complications Trial (DCCT) reported that intensive therapy produced HbA1c values that were around 2% lower than standard therapy, among insulin-dependent patients [42]. Also, on the basis of the current study, it is not possible to know whether reductions in HbA1c would be sustained beyond one year. The United Kingdom Prospective Diabetes Study (UK PDS) found that intensive glycaemic control was associated with continuing reductions in the risk of complications, even though HbA1c converged to the level experienced by those receiving conventional therapy after the first year [43]. However, the short-term HbA1c differences detected by the UK PDS were larger than those in the current study, and were assessed at 12 months, rather than across a 12-month period as in the current study.

A final and important consideration is that, since the DCCT, research has suggested that the implications of tighter glycaemic control on patient outcomes are unclear, and furthermore depend on the patient [2,32,44]. Thus, although some meta-analyses have found that intensive glycaemic control is associated with lower rates of major cardiovascular events in trials [45-47], another has suggested that the harms associated with severe hypoglycaemia might counterbalance the potential benefits of intensive glycaemic control [48]. An observational study found a U-shaped relationship between HbA1c and survival, with more deaths occurring at the bottom as well as the top of the HbA1c distribution compared with at the middle (7.5% or 58.5 mmol/mol). Another observational study found a U-shaped relationship between HbA1c and self-reported hypoglycaemia [49].

In light of the complex relationship between achieved HbA1c levels and patient outcomes, decision-making should take account of other outputs from the Whole Systems Demonstrator evaluation, including the forthcoming analysis of disease-specific quality of life, and the existing outputs regarding poor overall cost effectiveness [21]. Future, longer-term studies could examine impacts of telehealth on complications of diabetes, such as retinopathy and acute myocardial infarction.

## Competing interests

There were no competing interests.

## Authors’ contributions

AS led the collection of routine data sets for this paper, conducted the analysis and drafted the manuscript. MB provided advice throughout. HD was statistical adviser, while ET conducted the background literature review and contributed towards the production of the manuscript while based at the Nuffield Trust. SN contributed to the manuscript and was Principal Investigator for the Whole Systems Demonstrator trial. All authors reviewed the manuscript. The authors collectively made the decision to submit the manuscript for publication. All authors read and approved the final manuscript.

## Pre-publication history

The pre-publication history for this paper can be accessed here:

http://www.biomedcentral.com/1472-6963/14/334/prepub

## Supplementary Material

Additional file 1 Contains information on the Read codes used to derive variables from the general practice data sets. Click here for file

Additional file 2 Contains sensitivity analysis to model specification. Click here for file
