# Supplementary material for: Effect of telehealth on glycaemic control: analysis of patients with type 2 diabetes in the Whole Systems Demonstrator cluster randomised trial
Source: BMC Health Serv Res. 2014 Aug 6;14:334. doi: 10.1186/1472-6963-14-334 (PMC4128403; doi:10.1186/1472-6963-14-334)
Supplement: Additional file 1 — Contains information on the Read codes used to derive variables from the general practice data sets. [file 1472-6963-14-334-S1.docx]

**Additional File 1**

The following Read codes (version 2) were used to define the variables used in this study:

| Diabetes | C10E.% (type 1)  C10F.% (type 2) |
| --- | --- |
| HbA1c | 44TB., 42W4., 42W5., 42WZ. |
| Insulin | f1…%, f2…%, fw…%, ph3..% |
| Sulphonylureas | f3…% |
| Metformin | fte..%, f41..%, ftb..%, ft7..%  ft4w.%, ft4v.%, ft4u.% |
| Thiazolidinediones | ft5..% |
| DPP 4 inhibitors | ft8..%, fta..% |
| Exenatide | ft9..% |
| Arcabose | ft1..% |
| Body mass index | 22K..% |
| Serum cholesterol | 44P..% |
| Ischaemic heart disease | G3... - G330z  G33z. - G3401  G342. - G366.  G38.. – G3z..  Gyu3.% |
| Current smoker | 1373.-1376., 137C., 137D., 137G., 137H., 137J., 137M., 137P.-137R., 137V., 137X.-137f., 137h. |
| Ex smoker | 1378., 1379., 137A., 137B., 137F., 137K., 137N., 137O., 137S., 137T., 137j., 137l. |
| Blood pressure | 246..% (excluding 2460., 2468., 246H., 246I., 246K., 246L., 246M.) |
