# Supplementary material for: Effect of telehealth on glycaemic control: analysis of patients with type 2 diabetes in the Whole Systems Demonstrator cluster randomised trial
Source: BMC Health Serv Res. 2014 Aug 6;14:334. doi: 10.1186/1472-6963-14-334 (PMC4128403; doi:10.1186/1472-6963-14-334)
Supplement: Additional file 2 — Contains sensitivity analysis to model specification. [file 1472-6963-14-334-S2.docx]

**Additional File 2**

We conducted a range of sensitivity analyses to test the robustness of the effect sizes for HbA1c to alternative model specifications.

*Comparison of average HbA1c before and after recruitment*

The analysis in the main manuscript summarised multiple HbA1c readings recorded for each individual during the trial period using the mean.

1. We examined the impact of summarising HbA1c readings using the median or the maximum, rather than the mean. Effect sizes were similar to those obtained using the mean (adjusted differences -0.33% and -0.29%, respectively, with p = 0.004 and 0.034).
2. We examined the impact of using the last HbA1c reading recorded for each individual during the trial period as the endpoint, rather than the mean. Last HbA1c readings occurred on average around 8 months into the trial period (65% into the trial period for controls, and 68% for intervention patients), versus around 6 months for across all readings on average (48% and 50%). Effect sizes were similar to those obtained using the mean HbA1c reading as the endpoint (adjusted difference -0.29%, with p = 0.025).
3. Had the adjustment been based on mean rather than last recorded HbA1c prior to recruitment, effects would have been slightly smaller but still close to statistically significant (adjusted difference -0.21%, 95% CI, -0.44% to 0.00%, p = 0.053).
4. Finally, we conducted a difference-in-difference analysis, using the mean to summarise both multiple HbA1c readings recorded in the year before the trial and multiple readings recorded in the trial period. This analysis used a linear model as before, but with fixed effects for intervention status and trial period. We estimated the effect of telehealth on HbA1c using the interaction between intervention status and period. No additional adjustment for baseline variables was done, though random effects were also included for general practice as before. Effect sizes were smaller than in the main analyses, and not statistically significant (-0.20%, -0.58% to 0.18%, p = 0.297).

*Proportions of patients below thresholds*

The analysis in the main manuscript compared intervention and control groups in terms of the proportion of patients with mean HbA1c under the 7.5% threshold that was targeted by general practices.

1. We examined the impact of using the last HbA1c reading recorded during the trial period, rather than the mean over the trial period. Using this approach, 35.1% of control patients were under the 7.5% threshold, and 41.0% of intervention patients. This corresponded to an unadjusted odds ratio of 1.33 (95% CI, 0.85 to 2.09, p = 0.209), or an adjusted odds ratio of 1.45 (95% CI, 0.90 to 2.33, p = 0.126).
2. We also conducted difference-in-difference analysis using the proportions with mean HbA1c less than 7.5% pre-trial and the proportions with mean HbA1c less than 7.5% within the trial period. This produced an unadjusted odds ratio of 1.46 (95% CI, 0.80 to 2.65, p = 0.219) and an adjusted odds ratio of 1.49 (95% CI, 0.81 to 2.76, p = 0.203).
